# Evaluation of the Rational Use of Broad-Spectrum Antibiotics According to Regulatory Guidelines in Hospitalized Patients: A Descriptive Study

**DOI:** 10.5812/ijpr-163289

**Published:** 2025-07-27

**Authors:** Alireza Kananinambani, Majid Shohrati, Mahdi Bagheri, Bita Najafian

**Affiliations:** 1 Faculty of Pharmacy, Baqiyatallah University of Medical Sciences, Tehran, Iran; 2 Department of Clinical Pharmacy, Faculty of Pharmacy, Baqiyatallah University of Medical Sciences, Tehran, Iran; 3 Department of Pediatrics, Faculty of Medicine, Baqiyatallah University of Medical Sciences, Tehran, Iran

**Keywords:** Antimicrobial Stewardship, Broad-Spectrum Antibiotics, Rational Antibiotic Use, Hospital Epidemiology, Microbial Culture, Automatic Stop Order, Antimicrobial Resistance, Hospital-Acquired Infections

## Abstract

**Background:**

Antimicrobial resistance (AMR) is becoming a serious issue for global health, with predictions of up to 10 million deaths each year by 2050. Antimicrobial stewardship programs (ASPs), supported by WHO, CDC, and various health organizations, are designed to improve antibiotic use through guidelines and reviews.

**Objectives:**

The present study examined adherence to national guidelines for broad-spectrum antibiotics in patients at Baqiyatallah Hospital in Tehran.

**Methods:**

Between November 2024 and March 2025, researchers analyzed 120 hospitalized patients at Baqiyatallah Hospital who were prescribed specific broad-spectrum antibiotics, such as meropenem and vancomycin. They collected information about the patients, lab results, culture findings, and their antibiotic prescriptions.

**Results:**

We found that only 33.3% of patients had a consult from infectious diseases (ID) specialists, and just 30% had their prescriptions reviewed by a pharmacist. Cultures were taken from 87.5% of patients. The ID specialists made continuation/discontinuation decisions in only 4 of 184 cases (2.2%).

**Conclusions:**

Overall, the use of broad-spectrum antibiotics at this hospital often deviates from the recommended guidelines. Involvement from ID teams and routine checks by pharmacists were not utilized as much as needed. These results indicate a clear need to improve ASPs and increase the involvement of clinical pharmacists to encourage better antibiotic use.

## 1. Background

Antimicrobial resistance (AMR) is a serious global problem. Without swift action, drug-resistant infections could lead to 10 million deaths annually by 2050 (1). In 2019, the CDC reported over 2.8 million resistant infections and approximately 35,000 deaths each year in the U.S. alone (2). In Iran and similar regions, antibiotic use is exceedingly high, with nearly 75% of ICU patients receiving antibiotics (3). From 2000 to 2016, overall use increased from 33.6 to 60 defined daily doses (DDD) per 1,000 people per day. This overuse of broad-spectrum antibiotics like carbapenems, colistin, and linezolid is contributing to increased drug resistance in bacteria such as *Enterococcus*, *Klebsiella*, and *Acinetobacter*, leading to serious health issues (4).

Antimicrobial stewardship programs (ASPs) have become crucial in managing antibiotic use and combating the growing problem of AMR. Strong ASPs are increasingly seen as a key component of quality healthcare in hospitals and clinics (5). These programs aim to ensure patients receive the right medication, dose, and treatment duration (6, 7). Key actions include audits, pre-approvals, and regular reviews of ongoing treatments (8, 9). For instance, guidelines from IDSA/SHEA and the CDC recommend that doctors reevaluate all broad-spectrum antibiotics within 48 to 72 hours and avoid long-term use unless supported by lab evidence (10, 11). Studies show that effective stewardship can reduce antibiotic use without negatively impacting patient care (12). In one ICU study, these efforts reduced the number of days patients were on broad-spectrum antibiotics by about 20% and led to shorter hospital stays without increasing mortality rates. Conversely, antibiotic misuse can cause serious side effects in around 20% of patients and contributes to antibiotic resistance (13).

Iran faces significant challenges in managing antibiotic use. Research indicates that approximately 70 - 75% of ICU patients receive antibiotics, often without testing to confirm the appropriate choice or adjusting treatment based on results (14). The heavy use of drugs like carbapenems, vancomycin, and colistin is leading to increased resistance in bacteria such as *Acinetobacter baumannii*, *Klebsiella pneumoniae*, and vancomycin-resistant *Enterococcus* (1). To address this, Iran has initiated national programs to improve antibiotic stewardship, following guidelines from organizations like the CDC, IDSA, and WHO. One major benefit of AMS is its ability to reduce antibiotic overprescription (15). Research shows these programs can decrease the use of broad-spectrum antibiotics when a more targeted option is appropriate and help shorten antibiotic treatment durations to guideline recommendations (16). This targeted approach prevents unnecessary antibiotic use, helping to slow the growth of antibiotic-resistant bacteria (17).

Using ASPs typically reduces problems associated with antibiotics. Broad-spectrum antibiotics can disrupt beneficial bacteria in our bodies and increase the risk of issues like *Clostridioides difficile* infections. By promoting the use of more targeted antibiotics when appropriate, ASPs help maintain microbiome balance and reduce these risks, enhancing patient safety (18). Monitoring efforts allow us to assess stewardship effectiveness, identify areas needing improvement, and compare performance with other institutions. Tracking and providing feedback are crucial for the long-term success of ASPs (19).

A significant advantage of ASPs is their role in combating antibiotic resistance. By selecting the right antibiotics, dosages, and treatment durations, these programs help reduce the likelihood of resistant germs thriving. Monitoring antibiotic use and tracking resistance trends — common ASP practices — provide valuable information to guide local prescribing and infection control, helping to prevent resistance spread (20).

In Iran, antibiotic management programs are still developing. A 2019 report indicated that switching from meropenem to other antibiotics was safe without affecting recovery rates (21). Another study from Tehran found that a carbapenem-focused program reduced costs without increasing mortality rates (22). However, the extent of guideline adherence in practice remains unclear. Therefore, we investigated broad-spectrum antibiotic use at Baqiyatallah Hospital, a teaching hospital in Tehran, by assessing adherence to stewardship rules, such as timely culture collection, specialist consultations, and automatic stop orders. There is limited data on guideline adherence in Iranian tertiary care hospitals. This study aims to evaluate compliance with guidelines for monitoring broad-spectrum antibiotic use in ICU patients at Baqiyatallah Hospital, ultimately determining the extent of microbial culture and the determination of the antibiotic prescription route by the infectious disease (ID) specialist.

## 2. Objectives

The present study primarily aimed to evaluate compliance with antibiotic stewardship guidelines for broad-spectrum agents in hospitalized patients at Baqiyatallah Hospital and secondarily to identify patterns of culture use, ID specialist/pharmacist consultation, and prescribing practices affecting guideline adherence.

## 3. Methods

### 3.1. Study Design and Setting

We conducted a descriptive cross-sectional study at Baqiyatallah Hospital, Tehran (ICU-3 and CBRNE wards), from November 2024 to March 2025. Baqiyatallah is a tertiary care hospital where recently implemented stewardship guidelines restrict the use of select broad-spectrum antibiotics (meropenem, imipenem, vancomycin, colistin, linezolid, tigecycline, voriconazole, etc.) (Appendix 1 in Supplementary File).

### 3.2. Patients 

We screened inpatients who were prescribed any of the restricted broad-spectrum antibiotics during the study period. Inclusion criteria were hospitalization in target wards and receipt of at least one guideline-listed antibiotic. Exclusion criteria included immediate discontinuation (< 48 h) for non-infection reasons, patient refusal to participate, or incomplete records.

### 3.3. Data Collection

Demographic data (age, sex), clinical data (ward, admitting diagnosis, vital signs), and antibiotic treatment details were recorded for each patient. We used a standardized data collection form based on the Ministry of Health stewardship checklist, completed by reviewing medical records and patient interviews. Variables included: Antibiotic name(s) and dosing, duration of therapy, microbiological cultures ordered (blood, urine, sputum, wound, secretions), culture results (pathogens, susceptibilities), laboratory markers (WBC, CRP, creatinine, BUN, etc.), presence of ID specialist or clinical pharmacist consultation, and prescriber specialty. We noted if therapy was empiric versus culture-guided, and whether an “Automatic Stop Order” (pre-set discontinuation by day 5) was properly implemented.

### 3.4. Statistical Analysis

Data were entered into SPSS (version 26). Continuous variables are reported as mean ± SD or median (IQR). Categorical data are shown as counts and percentages. Normality was assessed by Kolmogorov-Smirnov tests. Group comparisons used one-way ANOVA (with Tukey post-hoc) or chi-square tests as appropriate. A P-value < 0.05 was considered statistically significant.

## 4. Results

### 4.1. Patient Characteristics

The study included 120 patients receiving broad-spectrum antibiotics. The mean age was 70.0 ± 12.8 years (range 20 - 91). The majority (85%) were in the CBRNE ward (102/120), with the remainder in ICU-3 (15%). Seventy patients (58.3%) were male. Specialists (non-ID) prescribed therapy in 63.3% of cases, while subspecialists prescribed in 36.7%. Only 40 patients (33.3%) had an ID consultation, and 36 (30.0%) had a clinical (pharmacist) consultation during their antibiotic course (Table 1). 

**Table 1.** Patient Demographics and Clinical Characteristics (N = 120) ^a^

Characteristics	Patients
**Sex (male) **	70 (58.3)
**ID specialist consultation**	40 (33.3)
**Clinical pharmacist consultation**	36 (30.0)
**Specialist prescriber (non-ID specialist)**	76 (63.3)
**Subspecialist prescriber**	44 (36.7)

Abbreviation: ID, infectious disease.

^a^ Values are expressed as No. (%).

Laboratory markers on presentation showed elevated inflammatory markers, with a mean WBC of 12.2 × 10^3^/µL and CRP of 100 mg/L. Creatinine levels changed significantly during treatment in 55% of patients, necessitating dose adjustments (e.g., meropenem, vancomycin) in 57.5% of cases.

### 4.2. Microbiological Cultures

At least one culture was obtained in 105 patients (87.5%) and total cultures were perfirmed. The distribution of requested and positive cultures is shown in Table 2. Blood cultures were ordered for 84 patients (70%), with 15 positives (17.9%), most commonly identifying *Enterococcus faecalis* (7 cases) and *Klebsiella pneumoniae* (5 cases). Urine cultures (n = 34) yielded pathogens in 9 cases (26.5%); sputum cultures (n = 58) were positive in 11 cases (19.0%). Wound and body fluid cultures were less frequent but predominantly positive, with *Acinetobacter* found in secretions. Overall, *Enterococcus*, *Klebsiella*, and *A. baumannii* were the dominant organisms identified.

**Table 2.** Microbiological Culture Results (N = 120) ^a^

Culture Type	No. Requested	Positive	Negative
**Blood**	84	15 (17.9)	69 (82.1)
**Urine **	34	9 (26.5)	25 (73.5)
**Sputum **	58	11 (19.0)	47 (81.0)
**Wound/fluid **	26	12 (46.2)	14 (53.8)
**Total **	202	47 (23.3)	155 (76.7)

^a^ Values are expressed as No. (%).

### 4.3. Antibiotic Prescribing Patterns

**Figure 1.**
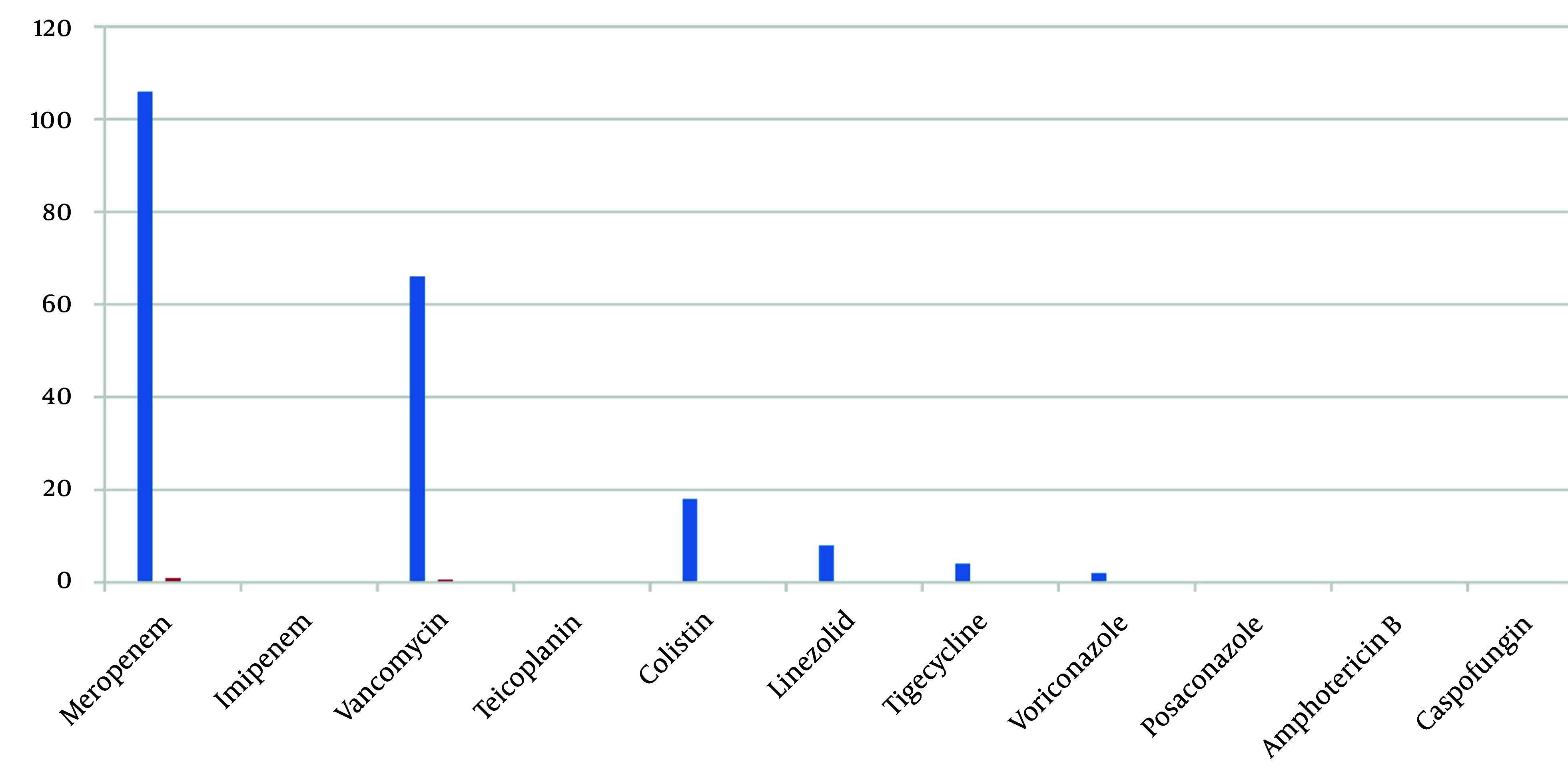
Use of broad-spectrum antibiotics by order of administration (N = 120)

**Figure 2.**
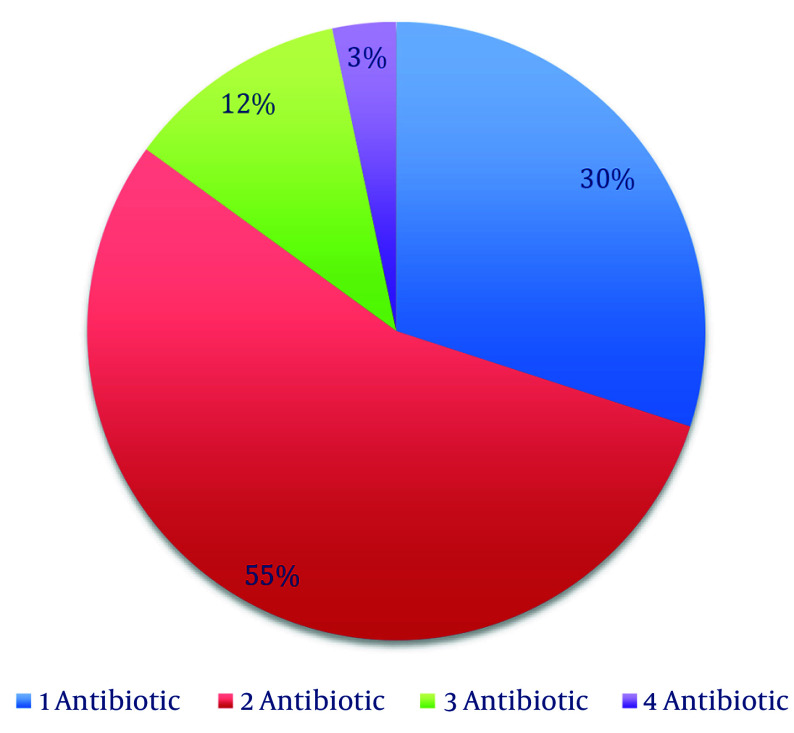
Percent of number of broad-spectrum antibiotics by order of administration (N = 120)

Broad-spectrum antibiotics prescribed (per stewardship list) included meropenem, vancomycin, colistin, linezolid, tigecycline, and voriconazole. Meropenem was the most common first-antibiotic: One hundred six patients (88.3%) received meropenem on days 1 - 4, whereas vancomycin was first-antibiotic in 12 patients (10.0%) (Figure 1). As additional therapy, vancomycin was added for 54 patients (45.0%) and colistin for 4 patients (3.3%). Linezolid (n = 8, 6.7%) appeared only as a second antibiotic. Third-antibiotic included colistin (n = 12, 10.0%) and tigecycline (n = 2, 1.7%). Fourth- antibiotic (rare) were voriconazole (n = 2, 1.7%) or tigecycline (n = 2, 1.7%). In summary, 66 patients (55.0%) received two broad-spectrum drugs, 14 (11.6%) received three, and 4 (3.3%) received four (Figure 2). The average number of broad-spectrum antibiotics per patient was 1.7.

### 4.4. Stewardship Adherence and Outcomes

According to national ASP guidelines, broad-spectrum therapy may proceed unmodified for the first 5 days under the admitting physician, but continuation beyond day 5 requires reassessment by an ID specialist. In our cohort, 184 of 204 antibiotic courses (90.2%) extended beyond 5 days (Table 3). However, ID specialists made continuation/discontinuation decisions in only 4 of 184 cases (2.2%); attending/ICU physicians decided in 162 cases (88.0%), and the treating physician in 18 cases (9.8%) (Table 3). Clinical pharmacists participated in reviews of 63% of prolonged courses. Furthermore, the mandatory “Automatic Stop Order” mechanism — which should have automatically discontinued therapy at 7 - 14 days unless reauthorized — was applied correctly in only 50% of eligible cases. Of 120 patients, 60 (50.0%) had their antibiotics properly timed-out at 5 days, whereas 60 (50.0%) did not (Table 3). In practice, many courses continued without documented reassessment.

**Table 3.** Duration More than Five Days and Review Responsibility (N = 204 Antibiotic Courses)

Criteria	Percent
**Duration > 5 (d)**	90.2
**Decision-maker after 5 (d)**	
ICU/attending physician	88.0
ID specialist	2.2
Treating physician	9.8
**Automatic stop order**	50.0

Abbreviation: ID, infectious disease.

### 4.5. Summary of Key Results

- A significant amount of broad-spectrum antibiotics were used. Meropenem was administered to 88% of patients initially, often in combination with vancomycin (55% received both) or colistin (used in 25% of cases). This indicates that treatment was primarily based on empirical therapy rather than being tailored to individual needs.

- Only about a third of the cases involved an ID consultant, and very few long-term treatment decisions included ID specialists (just 2.2%). Additionally, only 30% of patients had a clinical pharmacist review their medications.

- Stewardship practices were not very strong. Despite guidelines, automatic stop orders were applied to only half of the patients, potentially leading to unnecessary extended treatments. While most patients had cultures performed, these did not significantly influence treatment management.

## 5. Discussion

This study indicates that our hospital is not effectively managing broad-spectrum antibiotics. Similar to other reports, we observed an overuse of broad-spectrum therapy, with meropenem being prescribed more frequently than other options. This mirrors a situation in an Indian ICU, where a 20% reduction in meropenem use was noted after implementing an ASP (23). We also found a high incidence of combined therapy, such as using meropenem and vancomycin together in 13.3% of cases when it was unnecessary. This suggests a tendency among doctors to over-prescribe antibiotics as a precaution. Over 86% of treatments exceeded five days, longer than recommended for most infections. Only a third of patients received consultations with ID specialists. Studies indicate that involving these experts leads to better antibiotic use and outcomes. In our data, only 2% of prolonged treatments were reviewed by an ID doctor. Ideally, ASP teams comprising various specialists should review all cases. Clinical pharmacists also play a crucial role in stewardship, but only 30% of our patients received input from them. Hospitals with joint ASP teams, including both doctors and pharmacists, tend to perform better in stewardship.

Almost 90% of patients had cultures taken, which is commendable compared to other places. However, obtaining culture results did not always lead to treatment adjustments. We primarily identified *Enterococcus* and *Klebsiella*, including some resistant strains, highlighting common pathogens in our setting. Guidelines suggest narrowing antibiotic use based on test results, such as using vancomycin specifically for MRSA. Our study found that broad-spectrum antibiotics often continued even after culture results were available, indicating an opportunity for improvement. Other studies have shown that using time-outs or stop orders can reduce unnecessary antibiotic use (24-26). At our hospital, only half of the orders had appropriate stop rules, suggesting many patients received more treatment than necessary. This aligns with warnings that poor management of stop orders can inadvertently extend treatment durations.

International guidelines emphasize the importance of a comprehensive ASP. Our hospital’s policy includes several key features like preauthorization, audits, and stop-orders, but implementation needs improvement. For instance, the CDC recommends regular audits and feedback, while the SHEA/IDSA guidelines stress the importance of collaboration between ID specialists and pharmacists. Successful programs often involve team rounds, restrictions on certain medications, and continuous education (27). In resource-limited settings like Iran, challenges such as limited staff and diagnostic tools can hinder effective programs. However, even simple measures — like mandatory ID consultations for certain drugs and setting stop dates for all broad-spectrum antibiotics — have proven effective in other settings. For example, a policy to automatically stop vancomycin after 48 hours in a children’s hospital reduced the number of treatments exceeding that time from 33% to 23%, demonstrating that enforcing policies can significantly change behavior (28).

Our findings highlight the need to strengthen ASPs at Baqiyatallah Hospital. Here are some steps we can take: (1) Mandate ID specialist reviews of all restricted antibiotics by day 3 - 5 to adjust treatment; (2) have pharmacists regularly check dosing and kidney function adjustments — our study found that 6.7% had dosing errors related to kidney issues, consistent with other studies showing pharmacist involvement is beneficial; (3) implement automatic stop orders: Every antibiotic prescription should have a set end date unless renewed by ID, in line with best practices; (4) provide ongoing feedback: Regularly track antibiotic use and resistance data and share it with clinicians to encourage changes, as successful ASP advocates do.

We should note some limitations of our study: It is from a single center and is observational, so we cannot confirm cause and effect. Additionally, we did not directly measure clinical outcomes like infection cure or death rates, but previous studies suggest that ASPs do not lead to worse outcomes.

### 5.1. Conclusions

At this hospital in Iran, broad-spectrum antibiotics were used more than necessary, and adherence to guidelines was lacking. There is a critical need to involve ID specialists and pharmacists more actively and ensure compliance with automatic stop rules. Strengthening ASPs will help ensure antibiotics are used wisely, reducing resistance and improving patient care.

ijpr-24-1-163289-s001.pdf

## Data Availability

The data presented in this study are uploaded during submission.

## Clinical Trial Registration Code:

Project tracking code at Baqiyatallah University of Medical Sciences is 403000263.
