# Supplementary material for: Evaluation of the Rational Use of Broad-Spectrum Antibiotics According to Regulatory Guidelines in Hospitalized Patients: A Descriptive Study
Source: Iran J Pharm Res. 2025 Jul 27;24(1):e163289. doi: 10.5812/ijpr-163289 (PMC12523958; doi:10.5812/ijpr-163289)
Supplement: ijpr-24-1-163289-s001.pdf [file ijpr-24-1-163289-s001.pdf]

Appendix.1

جمهوری اسلامی ایران

وزارت بهداشت، درمان و آموزش پزشکی

معاونت درمان

فرم تجویز منطقی آنتی بیوتیک های وسیع الطیف

(Broad spectrum antibiotics stewardship form)

(نسخه دوم)

زمستان ۱۴۰۲

- نام داروها: ایمی پنم/ مروپنم- وانکومایسین/ تیکوپلانین - کلیستین- لینزولید- کسپوفانژین- وریکونازول- پوساکونازول- آمفوتریسین(داکسی کولات (معمولی)/ لیپومازول)- تیگسیکلین

- بخش درخواست کننده: تاریخ تجویز: تاریخ تکمیل فرم:

<table>
<tr><td colspan="6">اطلاعات دموگرافیک بیمار</td></tr>
<tr><td>نام</td><td>نام خانوادگی</td><td colspan="2">شماره پرونده</td><td>جنس:</td><td>سن:</td></tr>
<tr><td colspan="6">نتایج میکروبیولوژی (کشت):</td></tr>
<tr><td colspan="2">نوع میکروارگانیسم:</td><td colspan="2">نمونه:</td><td colspan="2">تاریخ ارسال نمونه:</td></tr>
<tr><td colspan="6">آنتی بیوگرام:
حساس:
مقاوم:</td></tr>
<tr><td colspan="6">علت قطع:
علت تغییر:
علت ادامه:</td></tr>
</table>

۴-پس از شروع یا ادامه آنتی بیوتیک، جهت نحوه تجویز و تنظیم دوز آنتی بیوتیک بر اساس محاسبات فارماکوکینتیکی و پارامترهای فارماکودینامیک، مشاوره با متخصص داروسازی بالینی (در صورت شاغل بودن یا در دسترس بودن در بیمارستان)، بر اساس نظر پزشک معالج، انجام شود.

۵- این فرم صرفا برای ۵ روز تکمیل میشود و درصورت نیاز به ادامه این آنتی بیوتیک باید فرم جدید، مجددا پر و تایید گردد.

۶-تحویل این آنتی بیوتیک ها از داروخانه با حضور مستقیم داروساز بالینی و یا داروساز شاغل در بیمارستان می باشد.

۷- پزشک معالج و پزشک مشاوره دهنده، مسئولیت تکمیل فرم را بر عهده دارند.

مهر و امضا پزشک معالج :

مهر و امضا متخصص عفونی و گرمسیری (بزرگسال /کودکان) مشاوره دهنده:

مهر و امضای داروساز بالینی/داروساز در زمان تحویل دارو:
